# Diagnostic Accuracy of Dried Blood Spots Collected on HemaSpot HF Devices Compared to Venous Blood Specimens To Estimate Measles and Rubella Seroprevalence

**DOI:** 10.1128/mSphere.01330-20

**Published:** 2021-07-14

**Authors:** Christine Prosperi, Ojas Kaduskar, Vaishali Bhatt, Alvira Z. Hasan, Jeromie W. Vivian Thangaraj, Muthusamy S. Kumar, R. Sabarinathan, Saravana Kumar, Augustine Duraiswamy, Gururaj Rao Deshpande, Ullas Padinjaremattathil Thankappan, Sanjay L. Chauhan, Ragini N. Kulkarni, Avi Kumar Bansal, Itta K. Chaaithanya, Neha R. Salvi, Sandeep Sharma, William J. Moss, Lucky Sangal, Nivedita Gupta, Manoj V. Murherkar, Sanjay M. Mehendale, Gajanan N. Sapkal, Kyla Hayford

**Affiliations:** a Department of International Health, International Vaccine Access Center, Johns Hopkins Bloomberg School of Public Health, Baltimore, Maryland, USA; b Diagnostic Virology Group, ICMR-National Institute of Virology, Pune, Maharashtra, India; c Indian Council of Medical Research (ICMR)-National Institute of Epidemiology, Chennai, India; d Department of Operational Research, Indian Council of Medical Research (ICMR)-National Institute for Research in Reproductive Health (NIRRH), Mumbai, Maharashtra, India; e Division of Epidemiology, ICMR-National JALMA Institute for Leprosy & Other Mycobacterial Diseases, Agra, India; f Department of Health Research, Model Rural Health Research Unit Dahanu, Sub District Hospital Compound, Dahanu, Maharashtra, India; g Department of Epidemiology, Bloomberg School of Public Health, Johns Hopkins University, Baltimore, Maryland, USA; h W. Harry Feinstone Department of Molecular Microbiology and Immunology, Bloomberg School of Public Health, Johns Hopkins University, Baltimore, Maryland, USA; i World Health Organization, Southeast Asia Region Office, New Delhi, India; j Division of Epidemiology & Communicable Diseases, Indian Council of Medical Research, New Delhi, India; University of Maryland School of Medicine

**Keywords:** dried blood spots, ELISA, India, measles, rubella, diagnostic accuracy, serology

## Abstract

Fingerprick blood spotted onto filter paper offers an alternative to venous blood for use in population-based surveillance because it is comparatively inexpensive, acceptable, and easy to manage in the field. Prior studies have shown excellent agreement for immunoglobulin G (IgG) antibody detection from dried blood spots (DBS) and venous blood samples. However, much of this evidence is from high-income settings or laboratories where the samples were unlikely to be exposed to extreme temperatures and humidity, factors known to degrade DBS. We report the diagnostic accuracy of DBS collected using HemaSpot HF devices against venous sera in measuring measles- and rubella-specific IgG antibodies in a household serosurvey conducted in two districts in India. Paired serum and DBS samples collected by fingerprick were collected from women aged 15 to 50 years enrolled in a serosurvey in Palghar District of Maharashtra and Kanpur Nagar District of Uttar Pradesh in India. Specimen quality and volume were assessed in the laboratory. Samples were tested for antimeasles and antirubella IgG antibodies by an enzyme-linked immunosorbent assay (ELISA) (Euroimmun). Sensitivity of antibody detection by DBS was greater than 98%, and specificity was 90% and 98%, for measles and rubella IgG, respectively. Antibody concentrations were strongly correlated between paired specimens with adequate volume (measles *R*^2^ = 0.94; rubella *R*^2^ = 0.89). Although correlation was poor if DBS specimens had lower volumes, impact on qualitative results was minimal. This study showed DBS collected with HemaSpot HF devices can generate highly accurate results of measles- and rubella-specific IgG compared to sera in community-based surveys when protocols are optimized for DBS specimens.

**IMPORTANCE** Dried blood spot (DBS) collection provides an easy, practical, and acceptable alternative to venous blood collection, especially for community-based studies, provided that results from DBS are accurate. We demonstrated high sensitivity and specificity for measles- and rubella-specific immunoglobulin G (IgG) with DBS collected via HemaSpot HF devices compared to serum samples. This is one of the largest community-based diagnostic accuracy studies of measles and rubella antibody testing with DBS and the first application we are aware of using HemaSpot HF device for measles and rubella serology. Results support the use of DBS in community-based serosurveillance.

## INTRODUCTION

Serosurveys can provide estimates of current, recent, or past exposure to infectious diseases in populations. Venous blood collection requires a trained phlebotomist, temperature-controlled sample transport to laboratories, and processing of samples soon after collection. As an alternative, population-based serosurveys can utilize dried blood spots (DBS) collected by fingerprick on filter paper to minimize the above-mentioned challenges in community-based surveys (1). While simplifying procedures in the field, antibody testing on dried blood spots can be more variable and less sensitive than venous blood samples (2–5).

Since the 1960s, DBS specimens have been routinely used for neonatal metabolic screening, and more recently, for HIV detection (1, 6, 7). Yet the diagnostic accuracy of DBS in measuring measles and rubella immunoglobulin G (IgG) by enzyme immunoassay is highly variable, showing sensitivity ranging from 96% to 100% and specificity from 75% to 100% (T. A. Holroyd et al., unpublished data). Variability is believed to be primarily driven by the quality of the DBS specimen, lack of standardized procedures in the laboratory when eluting sera from DBS, and the use of enzyme immunoassay not validated for use with DBS specimens (8).

Several researchers have demonstrated excellent qualitative agreement and quantitative concordance for measles IgG antibody detection with DBS compared to paired serum samples. Much of this evidence has been gathered in high-income settings, such as the United Kingdom and Australia, or in laboratory settings where the samples were unlikely to be exposed to extreme temperatures and humidity conditions, two factors known to impact the quality of a DBS specimen (5, 9–11). Most studies comparing paired venous blood and DBS samples for measles and rubella antibodies had relatively small numbers of participants and were most likely to use traditional filter papers (e.g., Whatman 903 cards) with limited evidence from newer DBS collection devices. Development of a measles- and rubella-specific IgG test that produces both quantitative and qualitative results that are reproducible and accurate compared to venous sera will be valuable for population-based serological surveillance. The goal of this study was to estimate the diagnostic accuracy of DBS collected on HemaSpot HF devices compared to venous blood samples for measuring measles and rubella-specific IgG antibodies in a population-based serosurvey carried out in two districts in India.

## RESULTS

**FIG 1:**
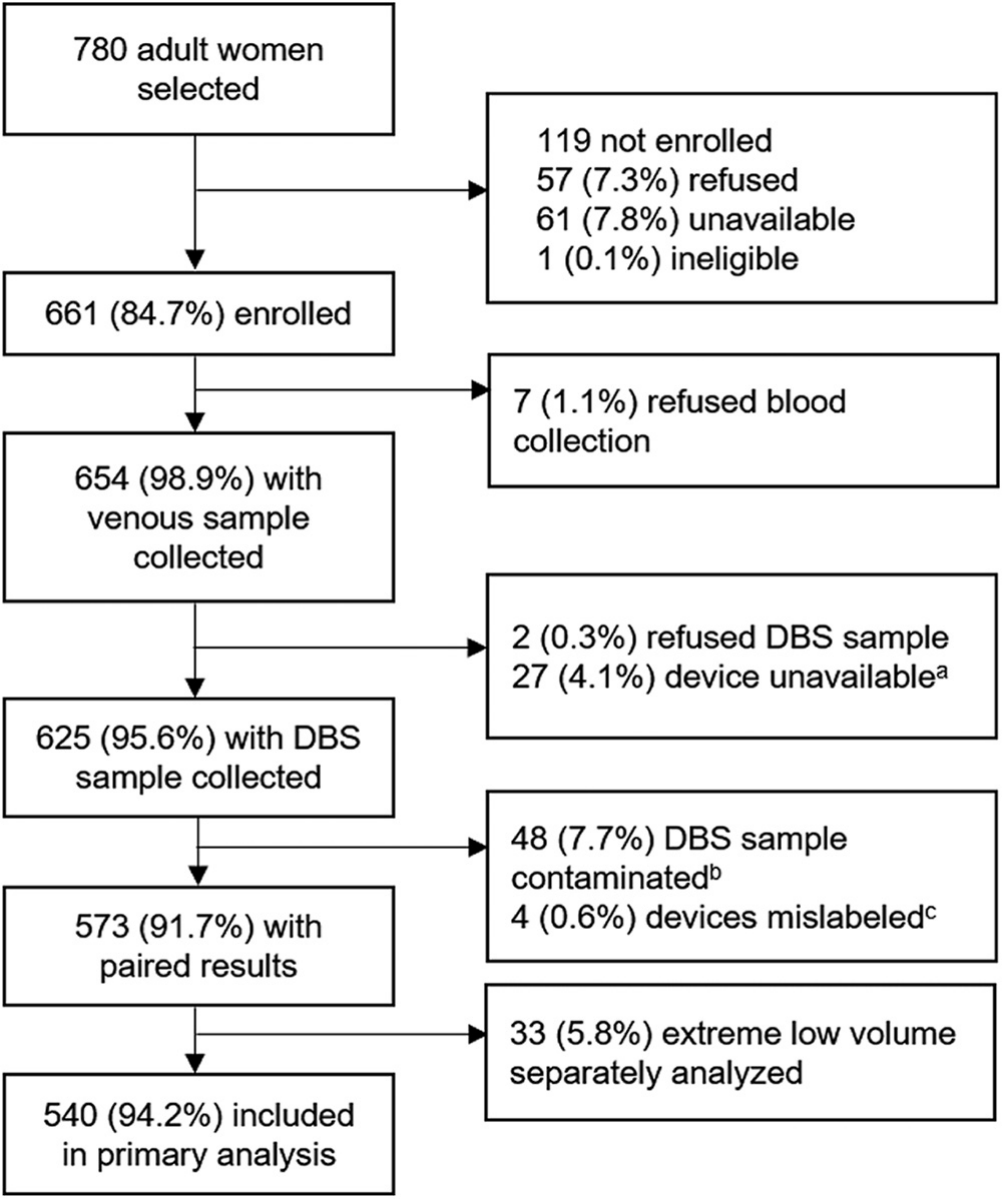
Flowchart of participants. Abbreviation: DBS, dried blood spot. Footnote a, device unavailable due to stock out. Footnote b, fungal or bacterial growth observed at the time of testing, primarily from one site. The collection protocol was revised to reduce contamination risk. Footnote c, four devices were mislabeled, and results were excluded from analyses.

Of the 780 women selected, 661 women were enrolled in the measles and rubella serosurveys in Palghar and Kanpur Nagar Districts in India. Seven percent of the eligible women refused enrollment (*n* = 57), of which two thirds (*n* = 39) reported blood collection as the reason for refusal. Among the enrolled women, 654 (98.9%) had venous blood collected and 625 of them (95.5%) had both venous and DBS sample collected (Fig. 1). Reasons for missing samples included refusal (*n* = 9, with 7 individuals refusing all blood collection and 2 accepting venous collection but refusing DBS collection) and unavailability of the DBS device due to a procurement delay at the Palghar site (*n* = 27, Fig. 1). The median age of the women participants was 29.7 years (interquartile range, 23.5, 38.0) (data not shown).

More than half of the DBS specimens (55%) had adequate volume in the HemaSpot HF device, and 34% had suboptimal volume (i.e., wedges incompletely saturated with blood), including 33 devices with extremely low volume (Table 1; see also Fig. S1 in the supplemental material). Eight percent of samples were excluded due to fungal or bacterial contamination and <1% due to mislabeling. Contamination was over three times higher in Palghar District, Maharashtra, India, probably due to its coastal location and nearly 100% humidity during the study period and where devices may have been exposed to the air for a longer duration than in Kanpur Nagar District, Uttar Pradesh, India, as described in Materials and Methods. Samples with contamination, mislabeling, or extreme low volume were excluded from subsequent analyses but secondary analyses, including devices with extremely low volumes are presented in the supplemental material. Consequently, we had 573 paired DBS-venous blood sample results available for the primary analysis (Table 1).

**TABLE 1:** Quality and volume of dried blood spot samples

DBS quality	Description	Included in primary analysis^*a*^	*n* (%)		
			All devices (*n* = 621)	Palghar district (*n* = 285)	Kanpur Nagar district (*n* = 336)
Adequate	All wedges fully saturated with blood	✓	343 (55.2)	143 (50.2)	200 (59.5)
Excessive volume	Oversaturation of wedges	✓	19 (3.1)	6 (2.1)	13 (3.9)
Low volume^*b*^	Incomplete saturation of wedges or uneven distribution of blood across wedges	✓	178 (28.7)	77 (27.0)	101 (30.1)
Extremely low volume^*b*^	Less than half of all the wedges were saturated or faint sample due to likely low analyte volume	X^*c*^	33 (5.3)	23 (8.1)	10 (3.0)
Contaminated	Fungal and/or bacterial growth on filter paper	X^*c*^	48 (7.7)	36 (12.6)	12 (3.6)

a A total of 625 DBS samples were collected, but 4 DBS samples were excluded due to labeling error.

b The wedge that was in the best condition compared to other wedges in the device was selected for testing. In a device with no completely filled wedge, the one that was visually the fullest was selected. In an unevenly distributed device, we selected the wedge that was completely full.

c Extremely low volume samples were tested and analyzed in secondary analyses. Contaminated samples were not tested.

10.1128/mSphere.01330-20.2 FIG S1 Images of HemaSpot devices by quality category. Download FIG S1, JPG file, 0.1 MB. Copyright © 2021 Prosperi et al. 2021 Prosperi et al. https://creativecommons.org/licenses/by/4.0/ This content is distributed under the terms of the Creative Commons Attribution 4.0 International license.

**FIG 2:**
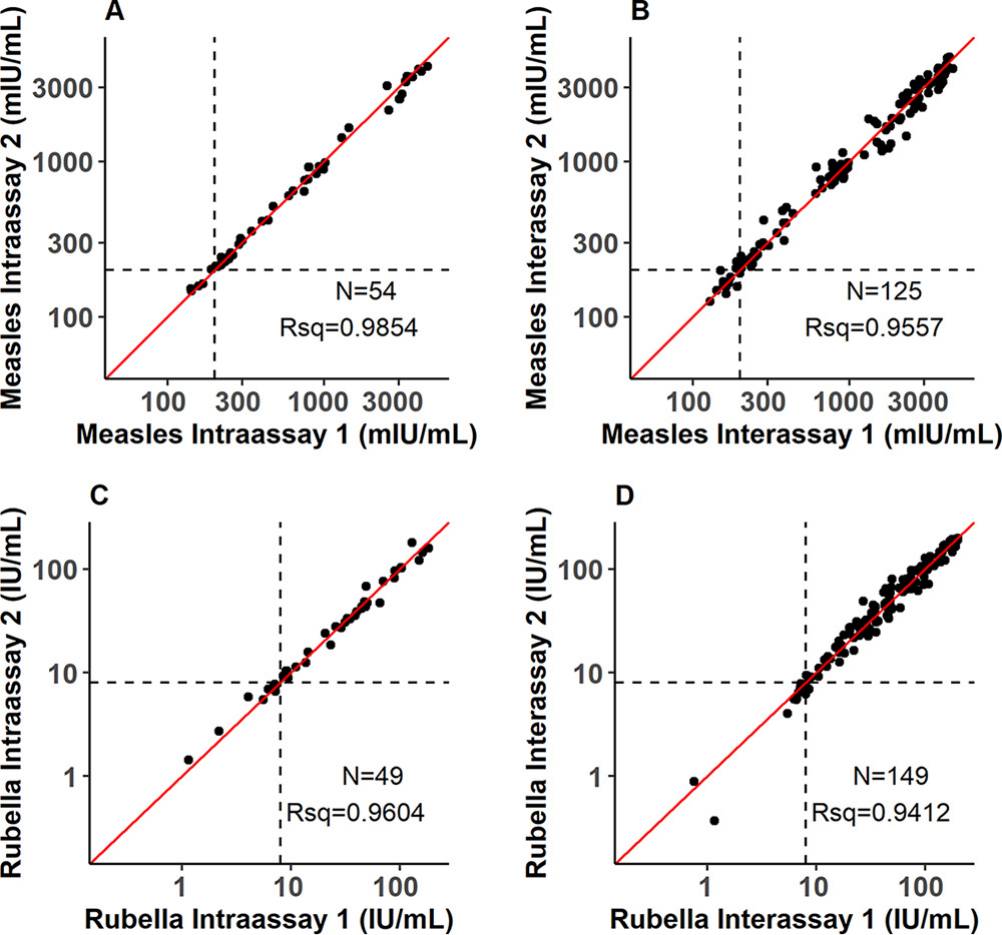
Intra-assay (A and C) and interassay (B and D) correlation of dried blood spot replicates, measles (top row) and rubella (bottom row). Contaminated, mislabeled, and extremely low volume samples were excluded. The red line indicates the line of equivalence. The dashed lines indicate equivocal thresholds (200 mIU/ml for measles and 8 IU/ml for rubella). Rsq, *R*^2^.

A wide range of antibody concentrations were observed: 51.4 to 20,225.8 milli international units (mIU)/ml for measles IgG and 0.0 to 499.0 IU/ml for rubella IgG. Intra- and interassay correlation of DBS replicates tested on the same plate (reflecting the same elution) and different plates (reflecting elution of two different wedges) were observed to be strong (*R*^2^ > 0.94) for both measles and rubella (Fig. 2).

### Qualitative agreement of DBS and sera.

Concordance between serum and DBS specimens was 98% for both measles and rubella (Tables 2 and 3). Of the few discordant results, two of three measles and all five rubella false-negative results had low DBS volume. Classifying equivocal results for both serum and DBS as positive, the sensitivity and positive predictive value (PPV) for measles IgG by DBS were 98.6% and 99.4%, respectively, and the specificity and negative predictive value (NPV) were 90.0% and 79.4%, respectively. For rubella IgG, the sensitivity and PPV of DBS were 98.4% and 99.8%, respectively, and the specificity and NPV were 98.1% and 86.4%, respectively, when equivocal results were classified as positive (Table 3). Excluding all equivocal results led to slightly higher sensitivity and eliminated false-positive results for DBS for measles and rubella.

**TABLE 2:** Qualitative concordance in measles and rubella antibody detection comparing paired serum and dried blood spot specimens^*a*^

Dried blood spot specimen result	No. of serum specimens							
	Measles				Rubella			
	Positive	Equivocal	Negative	Total	Positive	Equivocal	Negative	Total
Positive	492	1	0	493	474	1	0	475
Equivocal	4	6	3	13	4	1	1	6
Negative	3	4	27	34	5	3	51	59
Total	499	11	30	540	483	5	52	540

a Analysis excludes devices with contamination, extremely low volume, or mislabeling.

**TABLE 3:** Diagnostic accuracy in measles and rubella antibody detection comparing paired serum and dried blood spot specimens^*a*^

Parameter	% diagnostic accuracy (95% CI)			
	Treating equivocal results as positive results		Excluding equivocal results	
	Measles	Rubella	Measles	Rubella
Sensitivity	98.6 (97.2, 99.5)	98.4 (96.8, 99.3)	99.4 (98.2, 99.9)	99.0 (97.6, 99.7)
Specificity	90.0 (73.5, 97.9)	98.1 (89.7, 100)	100 (87.2, 100)	100 (93.0, 100)
PPV	99.4 (98.3, 99.9)	99.8 (98.9, 100)	100 (99.3, 100)	100 (99.2, 100)
NPV	79.4 (62.1, 91.3)	86.4 (75.0, 94.0)	90.0 (73.5, 97.9)	91.1 (80.4, 97.0)
% agreement	98.1	98.3	99.4	99.1
Kappa statistic	0.83 (0.73, 0.93)	0.91 (0.85, 0.97)	0.94 (0.88, 1.00)	0.95 (0.90, 0.99)

a Analysis excludes devices with contamination, extremely low volume, or mislabeling. 95% CI, 95% exact confidence intervals; PPV, positive predictive value; NPV, negative predictive value.

### Quantitative agreement of DBS and sera.

**FIG 3:**
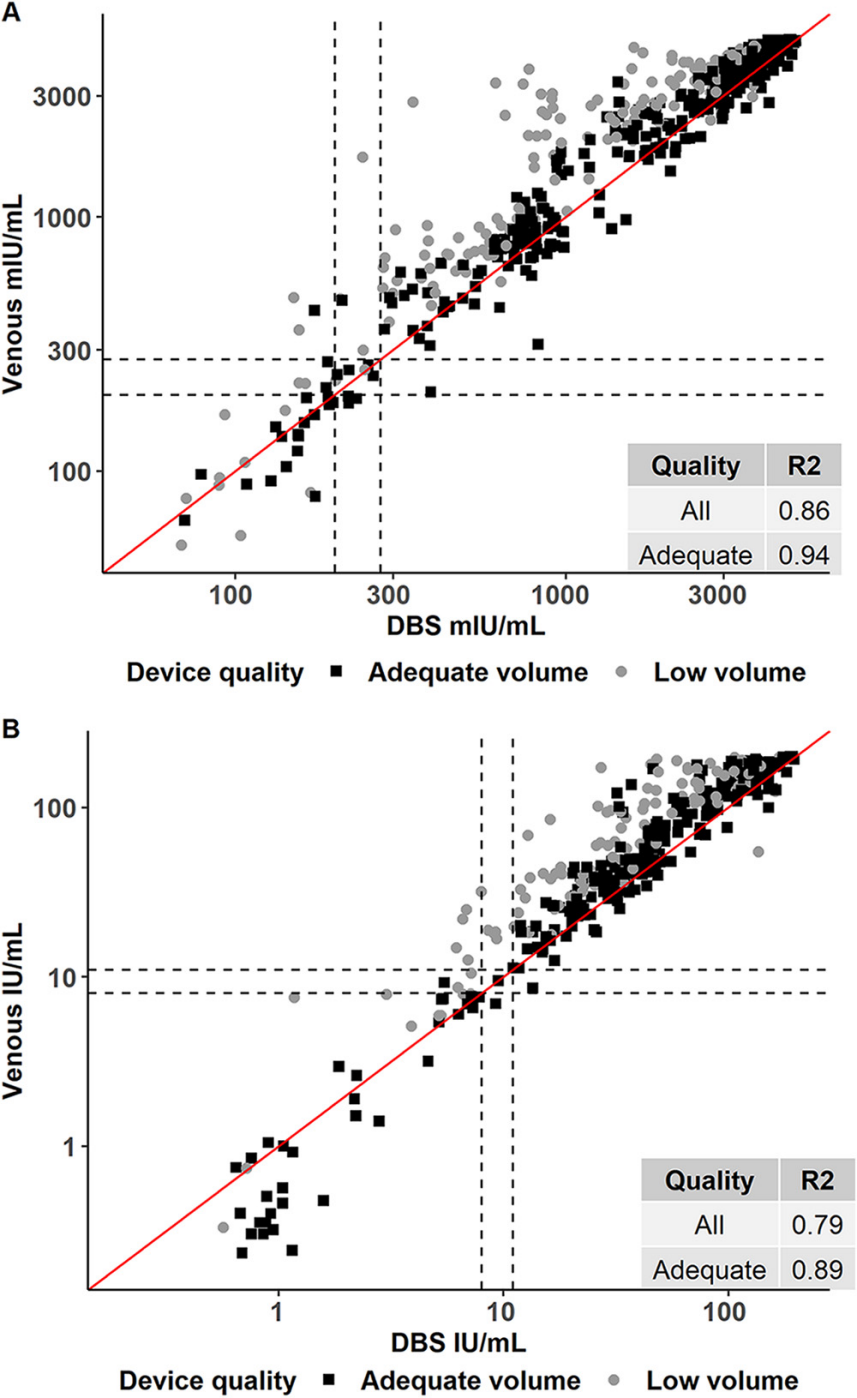
Quantitative relationship in measles (A) and rubella (B) antibody detected in serum versus dried blood spot specimen by quality of specimen. Graphs were restricted to values below the highest calibrator of assays (5,000 mIU/ml for measles and 200 IU/ml for rubella). The dashed lines indicate equivocal and positive thresholds (equivocal, 200 mIU/ml for measles and 8 IU/ml for rubella; positive, 275 mIU/ml for measles and 11 IU/ml for rubella). Excessive volume DBS samples were grouped with adequate volume samples for scatterplot but were excluded for the purpose of calculating the *R*^2^ among adequate samples (second row in inset box). The red line indicates the line of equivalence.

There was good correlation between the paired DBS-serum quantitative values for both measles (*R*^2^ = 0.86) and rubella (*R*^2^ = 0.79) across the range of antibody concentrations. On excluding specimens with low volume, correlation improved for measles (*R*^2^ = 0.94) and rubella (*R*^2^ = 0.89) (Fig. 3; see also Fig. S2). Despite variability in antibody concentrations, DBS volume had a small impact on the qualitative agreement apart from those classified as extremely low volume, which were excluded from the primary analysis and produced substantially lower antibody concentrations (Fig. 3 and Fig. S3).

10.1128/mSphere.01330-20.3 FIG S2 Quantitative relationship in measles (A) and rubella (B) antibody detected in serum versus dried blood spot specimen, restricted to adequate samples. Graphs restricted to values less than the highest calibrator (5,000 IU/liter for measles and 200 IU/ml for rubella). Dashed lines indicate equivocal and positive thresholds (equivocal, 200 mIU/ml for measles and 8 IU/ml for rubella; positive, 275 mIU/ml for measles and 11 IU/ml for rubella). The red line indicates the line of equivalence. Download FIG S2, TIF file, 0.8 MB. Copyright © 2021 Prosperi et al. 2021 Prosperi et al. https://creativecommons.org/licenses/by/4.0/ This content is distributed under the terms of the Creative Commons Attribution 4.0 International license.

10.1128/mSphere.01330-20.4 FIG S3 Quantitative relationship in measles (A) and rubella (B) antibody detected in serum versus dried blood spot specimen by DBS quality for all collected devices. Contaminated or mislabeled samples were excluded. Graphs restricted to values less than the highest calibrator (5,000 IU/liter for measles and 200 IU/ml for rubella). Dashed lines indicate equivocal and positive thresholds (equivocal, 200 mIU/ml for measles and 8 IU/ml for rubella; positive, 275 mIU/ml for measles and 11 IU/ml for rubella). The red line indicates line of equivalence. Download FIG S3, TIF file, 0.9 MB. Copyright © 2021 Prosperi et al. 2021 Prosperi et al. https://creativecommons.org/licenses/by/4.0/ This content is distributed under the terms of the Creative Commons Attribution 4.0 International license.

**FIG 4:**
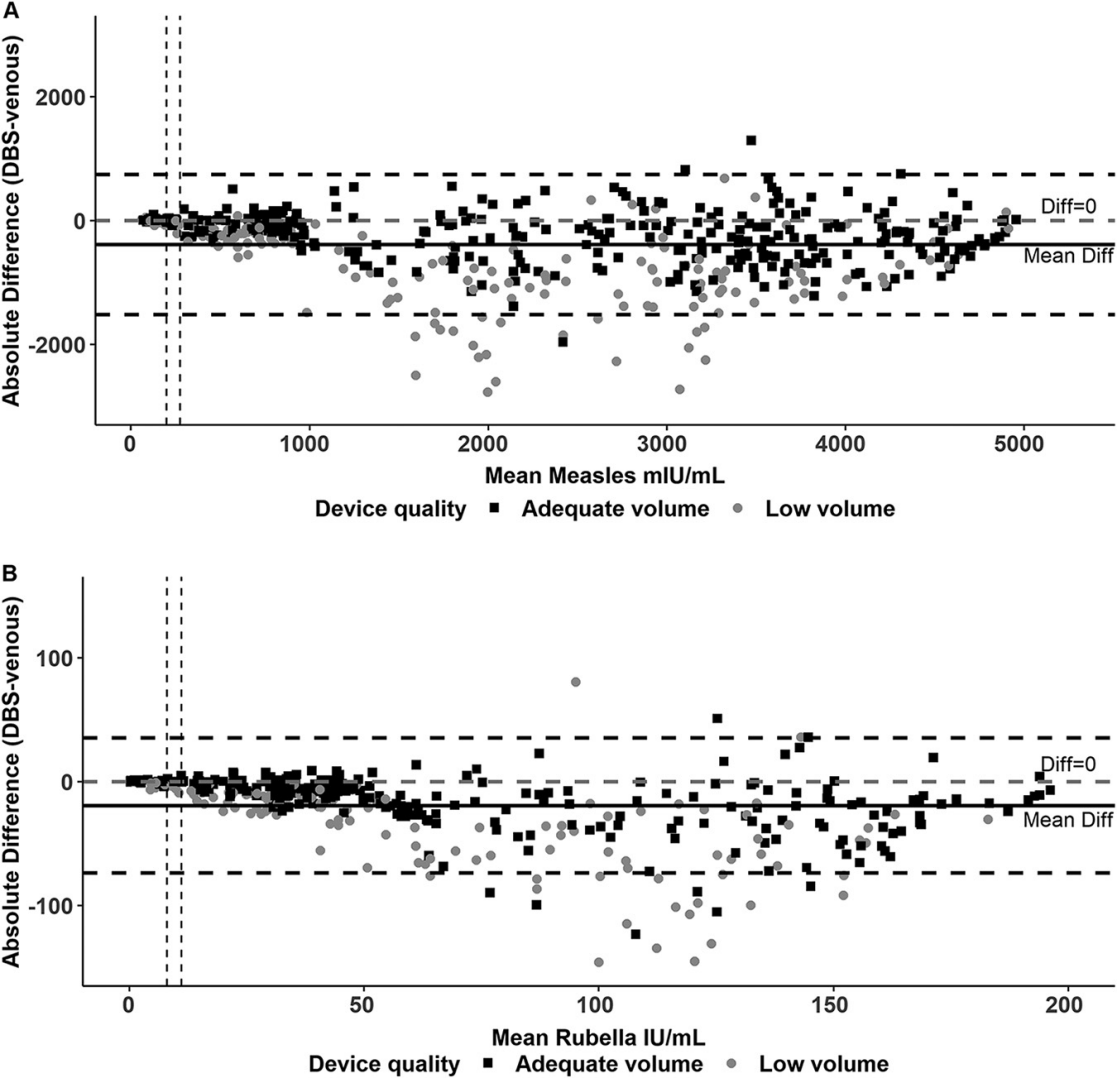
Mean of measles (A) and rubella (B) antibody concentrations plotted against the difference of serum versus dried blood spot. Vertical dashed lines indicate equivocal and positive thresholds (equivocal, 200 mIU/ml for measles and 8 IU/ml for rubella; positive, 275 mIU/ml for measles and 11 IU/ml for rubella). The gray dashed horizontal line indicates an absolute difference of 0. Black horizontal lines indicate the mean difference (solid line) and the limits of agreement (mean difference plus or minus 1.96 standard deviations of the difference; dashed lines). Excessive volume DBS samples were grouped with adequate volume samples.

Antibody concentrations were slightly lower in DBS than sera across the range of concentrations for both measles and rubella IgG (Fig. 4A and B). Most values were within the 95% limits of agreement with the exception of the mid-range positive specimens for both measles and rubella IgG. The majority of specimens below the lower limit of agreement were low-volume DBS specimens.

## DISCUSSION

We observed high diagnostic accuracy of DBS collected on HemaSpot HF devices for correctly classifying samples as seropositive and seronegative for measles and rubella IgG antibodies compared to venous blood samples. The sensitivity was greater than 98% for both rubella and measles IgG. The specificity was 98% for rubella and slightly lower (90%) for measles IgG. Misclassification occurred primarily among samples with concentrations near the threshold or among DBS samples with extremely low blood volumes. Antibody concentrations were strongly correlated between paired specimens. Although the correlation was poorer for those DBS specimens with low volume, volume had a negligible effect on the qualitative classifications of paired DBS and serum samples. The acceptability of DBS collection was high, and only few women who had given venous blood samples refused DBS sample collection. In this setting, acceptability for venipuncture was also high. Based on reports from survey staff, some women were more comfortable with venipuncture; however, this may be unique to India where blood collection by fingerprick is less common than in other settings.

Our findings are consistent with prior comparisons, which reported high sensitivity and correlation in antibody concentrations between DBS and sera (3–5, 9–13). We observed lower measles and rubella antibody concentrations in DBS specimens compared to sera, which was also consistent with other studies, though this was primarily observed for DBS specimens with low volume (i.e., wedges not fully saturated). Few papers report on the role of specimen quality in the concordance between DBS and sera, though Su et al. found that almost a quarter of Whatman 903 cards spotted directly from a fingerprick lacked at least one fully saturated blood spot (14).

This is one of the few studies to estimate measles and rubella IgG antibodies in a population-based survey using commercially available Euroimmun enzyme immunoassay (EIA) kits. Euroimmun has emerged as a commonly used EIA kit for measles and rubella IgG testing and is approved by the World Health Organization for measles and rubella surveillance. However, there is no standardized protocol recommended for testing DBS specimens with this kit. Optimization of the elution protocol and dilution ratios for DBS specimens is critical prior to specimen testing to generate accurate results from DBS (15, 16). In the absence of optimization, systematic biases might affect the results and their interpretation. The dilution ratios or wedge size may be adjusted during laboratory optimization, or a correction factor for quantitative results may be considered to address these biases (17, 18).

DBS specimen collection provides a valuable alternative to venous blood sample collection, especially during community-based studies in low-resource settings by eliminating the need for a cold chain in the short term and reducing specimen processing requirements, aside from overnight drying of DBS cards in a clean location. Because the HemaSpot HF device is closed and stored immediately, a data collector could work in remote locations for several days without access to refrigeration. A limitation for measles serology is that it is not currently possible to use DBS to measure neutralizing antibodies by microneutralization or plaque reduction neutralization (PRN) assays, which have been shown to be more sensitive than EIAs, especially among individuals with low positive antibody concentrations (19). These tradeoffs should be considered while choosing the specimen type during serosurveys, particularly for studies among infants with waning maternally derived antibodies or among previously vaccinated older children and adults.

Contamination and low blood volume are the two main factors affecting the quality of DBS samples, and these were the limitations of our study. Nearly 8% of samples were excluded due to contamination, which was primarily observed at the first study site. We hypothesized that contamination could have resulted from inadequate sterilization technique (e.g., blood passing over the fingernail before dropping into the device) and introduction of environmental or fungal contaminants while devices were left open; this is in contrast to risk of cross-specimen contamination with other filter papers such as Whatman 903 or TropBio. The devices were initially left open to dry for longer than the manufacturer recommended time of 1 to 2 min to facilitate drying in the environment of extreme humidity. Contamination rates declined substantially after revising the protocol to reduce the time for which the device was left open and emphasizing sterilization techniques. Although other studies left the HemaSpot device open for 5 to 10 min without reported contamination (20, 21), we recommend closing the devices within 1 to 2 min as recommended by the manufacturer. We believe that with these changes, future studies will have substantially lower contamination rates. Because none of the hypothesized causes of contamination were associated with measles or rubella susceptibility, it is unlikely that contamination could have introduced a bias in estimation of measles or rubella seroprevalence.

Volume is another key factor influencing specimen quality, which may disproportionately affect samples from younger children. In our experience, HemaSpot HF devices require a larger volume to fill the device and saturate all wedges than conventional DBS cards. With Whatman 903 or TropBio cards, blood is collected on separate spots on the card; therefore, it is possible to fully saturate several spots and leave others empty if blood volume is low. Given the design of the HemaSpot device, it is not possible to select and fully saturate specific wedges. We observed that 34% of DBS devices had wedges that were not fully saturated, which resulted in lower antibody concentrations compared to serum specimens. Inadequate pricking of the finger with the lancet or issues related to blood flow can also be important and need to be investigated. A study that used self-collection by fingerprick onto HemaSpot devices found that 28% of participants had trouble collecting sufficient blood (22). Additionally, we observed that the filter paper within some devices did not lay flat, with some wedges tilted either up or down, and this may have led to uneven distribution of blood on certain wedges resulting in partial saturation. This matter was discussed with the manufacturer after completion of the study, and it was concluded that this could have been due to lot issues.

This is one of the largest diagnostic accuracy studies of measles and rubella antibody testing with DBS using specimens collected in the community as opposed to controlled clinical settings. Our study was conducted just prior to or during the rainy season in these districts reporting high temperatures and high humidity. It is possible that different specimen-quality issues may be encountered in other settings such as desert-like areas with extreme dust. Our report is the first application we are aware of using HemaSpot HF device for measles and rubella IgG antibody detection. Studies have reported use of HemaSpot for HIV, hepatitis C virus seromarkers, and blood glucose (20–23). Though there are advantages with HemaSpot in terms of an integrated collection and storage device, less possibility of oversaturation, and ease of elution, the device is considerably more expensive than traditional devices, and there is still risk of contamination as we observed. A more detailed comparison of HemaSpot with other devices is described in Kaduskar et al. (15). Our findings on the importance of DBS volume and optimization of dilution ratios and elution protocols are relevant for all DBS devices. Given the importance of DBS volume on antibody concentrations, it is important to conduct rigorous training on specimen collection and to document and monitor estimated volume as well as specimen quality of DBS devices. Although this study was restricted to adult women, there was a wide variability in measles and rubella antibody levels, including negative results and low positive results. Future studies should explore whether the diagnostic accuracy is different among children or highly vaccinated populations.

Unlike many prior papers that compare DBS and serum specimens, we focused our analysis on factors in the field related to collection and transport hypothesized to affect diagnostic accuracy. We observed strong correlation and concordance between DBS and serum specimens, providing additional evidence for the use of DBS in community-based research. However, we recommend careful monitoring of the specimen quality and volume given its influence on antibody concentrations. We also recommend that studies using DBS record data on specimen quality and volume, which can be used to improve quality of procedures in the field and make adjustments in the laboratory and data analyses.

## MATERIALS AND METHODS

### Study setting and population.

Paired serum and DBS samples were collected from women aged 15 to younger than 50 years enrolled in serosurveys after the measles and rubella vaccination campaigns in Palghar District, Maharashtra, India, and Kanpur Nagar District, Uttar Pradesh, India. Data were collected during April to September 2019. Thirty villages or wards were selected from each district based on the 2011 nationwide census using probability proportional to size systematic sampling method. One census enumeration block (CEB) was randomly selected from the list of CEBs in each village or ward. Thirteen individuals were randomly selected from each of the following three age groups: children 9 months to younger than 5 years, children aged 5 to 15 years, and women aged 15 to 50 years. Paired DBS samples were collected only from adult women for logistical reasons. The surveys were planned by the Indian Council of Medical Research (ICMR) and Johns Hopkins Bloomberg School of Public Health and implemented by the Model Rural Health Research Units (MRHRUs) of Department of Health Research in both selected districts.

Written informed consents were obtained from all participants or the legal guardians of the participants aged less than 18 years. Assent was obtained from children aged 7 to less than 18 years.

### Sample collection and processing.

Up to 2 ml of venous blood was collected in a serum separator tube (catalog no. 367983; Becton Dickinson) from each survey participant. Samples were left at room temperature for 30 min after collection, centrifuged at 3,000 rpm for 10 min using a portable centrifuge (Medico Plus centrifuge; REMI, Mumbai, India; Eltek Multispin Centrifuge, Elektrocraft, Mumbai, India), and stored at 4 to 8°C in cold boxes until transported back to the laboratory at the end of the day. In the laboratory, specimens were recentrifuged, and sera were aliquoted and stored at −20°C.

Immediately following venous blood collection, the finger of every woman participant aged 15 to 50 years was pricked using a contact-activated retractable lancet (catalog no. 366594; Becton Dickinson, Franklin Lakes, NJ, USA), and droplets were collected directly from the finger onto HemaSpot HF devices (Spot On Sciences, San Francisco, CA, USA) until the device was fully saturated with approximately 150 μl of whole blood (see Fig. S1a in the supplemental material). In Palghar District, where the first survey with DBS was conducted, the HF devices were left open for approximately 2 to 5 min after collection to allow time for the capillary blood sample to absorb on the filter paper prior to closing. However, the procedure was changed to immediate closure of the device after blood collection following reports of contamination. This change occurred at the end of the Palghar District survey and beginning of the survey in the Kanpur Nagar District. Each closed DBS device was placed in a sealed Ziploc bag containing two desiccants and stored at room temperature (approximately 20 to 25°C) in airtight plastic containers, while its corresponding sera were stored in vials at −20°C. Serum and DBS samples were transported to the ICMR-National Institute of Virology (ICMR-NIV), Pune, India, on dry ice at −20°C (sera) or room temperature (DBS) within 4 weeks of sample collection. Device saturation, adequacy of sample volume, and absence of sample contamination were confirmed at the ICMR-NIV laboratory before serological testing.

### Specimen testing.

Paired serum and DBS samples were tested on the same plate using commercially available antimeasles IgG and antirubella IgG kits (Euroimmun, Perkin Elmer, Germany). We estimated each wedge of the HemaSpot device contained 9.3 μl of sera (15). Sera were processed according to the manufacturer’s protocol, diluting samples 1:101 prior to testing. DBS samples were eluted at ICMR-NIV as described elsewhere (15). In brief, one wedge was selected from each device and placed into a cryotube containing 100 μl elution buffer and incubated for 2 h at 37°C with agitation for 20 min before and after incubation. Based on extensive standardization testing for the optimal elution and dilution ratios, 30 μl of eluted DBS were diluted using 210 μl of Euroimmun ELISA kit diluent of both measles and rubella separately in a ratio of 1:8 for the DBS samples (15). All subsequent steps for ELISA testing were identical for sera and DBS. Results were interpreted qualitatively based on the manufacturer’s recommendations. For measles IgG antibodies, values of  ≥275 mIU/ml were classified positive, values of ≥200 to <275 were classified equivocal, and values of <200 were classified negative. For rubella IgG antibodies, values of  ≥11 IU/ml were classified positive, those with values of ≥8 and <11 were classified equivocal, and those with values of <8 were classified negative.

Samples equivocal for either measles or rubella were retested in duplicate, and the qualitative result most commonly observed out of the three results was selected as final. Measles samples above the top calibrator 1 (5,000 mIU/ml) were retested at a further fourfold dilution to obtain the final quantitative result. Rubella samples above the top calibrator (200 IU/ml) were not retested. Four randomly selected samples, either DBS or sera, were run in duplicate to monitor intraplate variability. Every 20th specimen per plate was retested to assess interplate variability.

### Data analysis.

Sample size was based on enrolling a sufficient number of participants negative for measles and rubella in serum to maximize precision around specificity, while accounting for operational feasibility. Sensitivity, specificity, positive predictive value (PPV), and negative predictive value (NPV) were calculated by comparing the qualitative results of paired DBS and sera, the reference specimen. Agreement was evaluated categorically using percent concordance and kappa, and quantitatively by *R*^2^ statistics. Systematic error across the range of antibody levels was assessed using Bland-Altman plots (24). All analyses were performed using SAS statistical software (version 9.4; SAS, Cary, NC, USA) and R (version 3.4.4, Vienna, Austria).

10.1128/mSphere.01330-20.1 TABLE S1 Qualitative concordance in measles and rubella antibody detection comparing paired serum and dried blood spot specimens (including extremely low volume samples). Assay parameters were calculated treating equivocal results as positive results. Note that the analysis excludes devices with contamination, extremely low volume, or mislabeling. 95% exact confidence intervals are shown. Download Table S1, DOCX file, 0.03 MB. Copyright © 2021 Prosperi et al. 2021 Prosperi et al. https://creativecommons.org/licenses/by/4.0/ This content is distributed under the terms of the Creative Commons Attribution 4.0 International license.
