# Supplementary figures and images for: Diagnostic Accuracy of Dried Blood Spots Collected on HemaSpot HF Devices Compared to Venous Blood Specimens To Estimate Measles and Rubella Seroprevalence
Source: mSphere. 2021 Jul 14;6(4):e01330-20. doi: 10.1128/mSphere.01330-20 (PMC8386429; doi:10.1128/mSphere.01330-20)

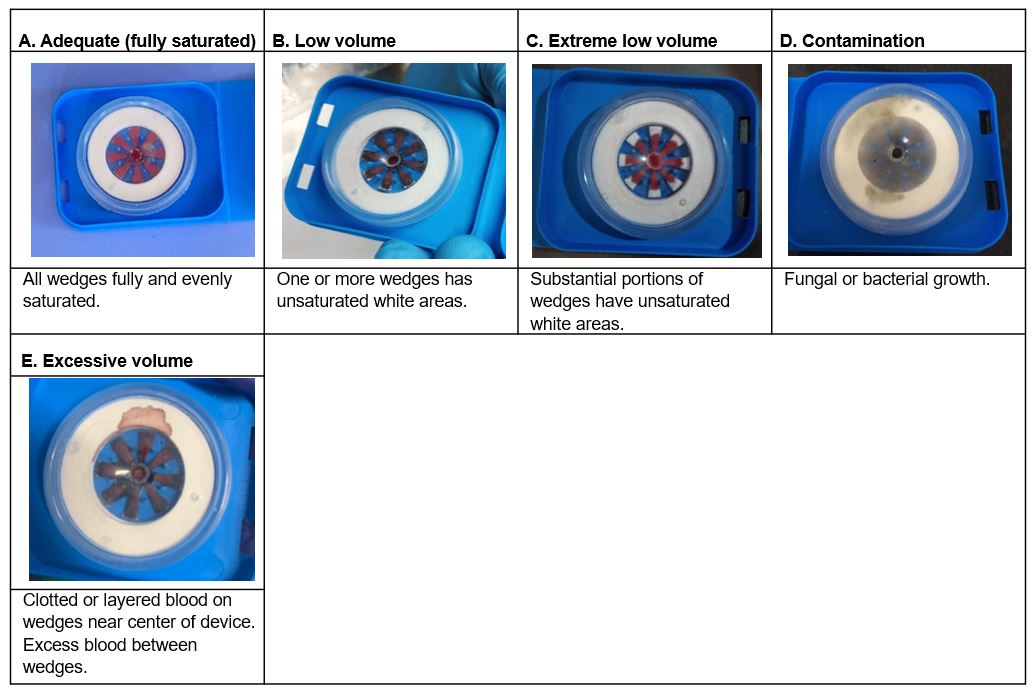

Supplement: FIG S1 [file msphere.01330-20-sf001.jpg]

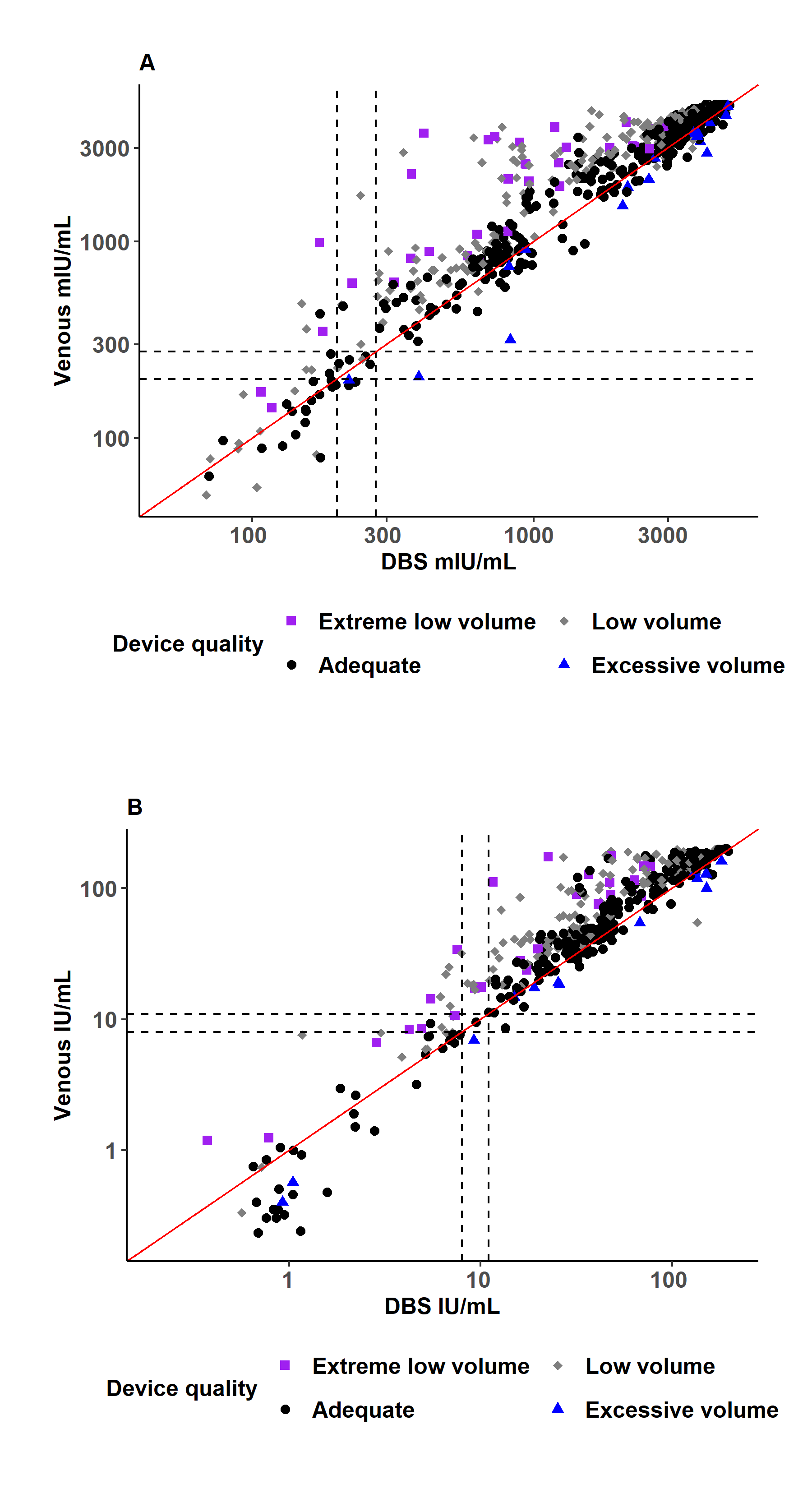

Supplement: FIG S2 [file msphere.01330-20-sf002.tif]

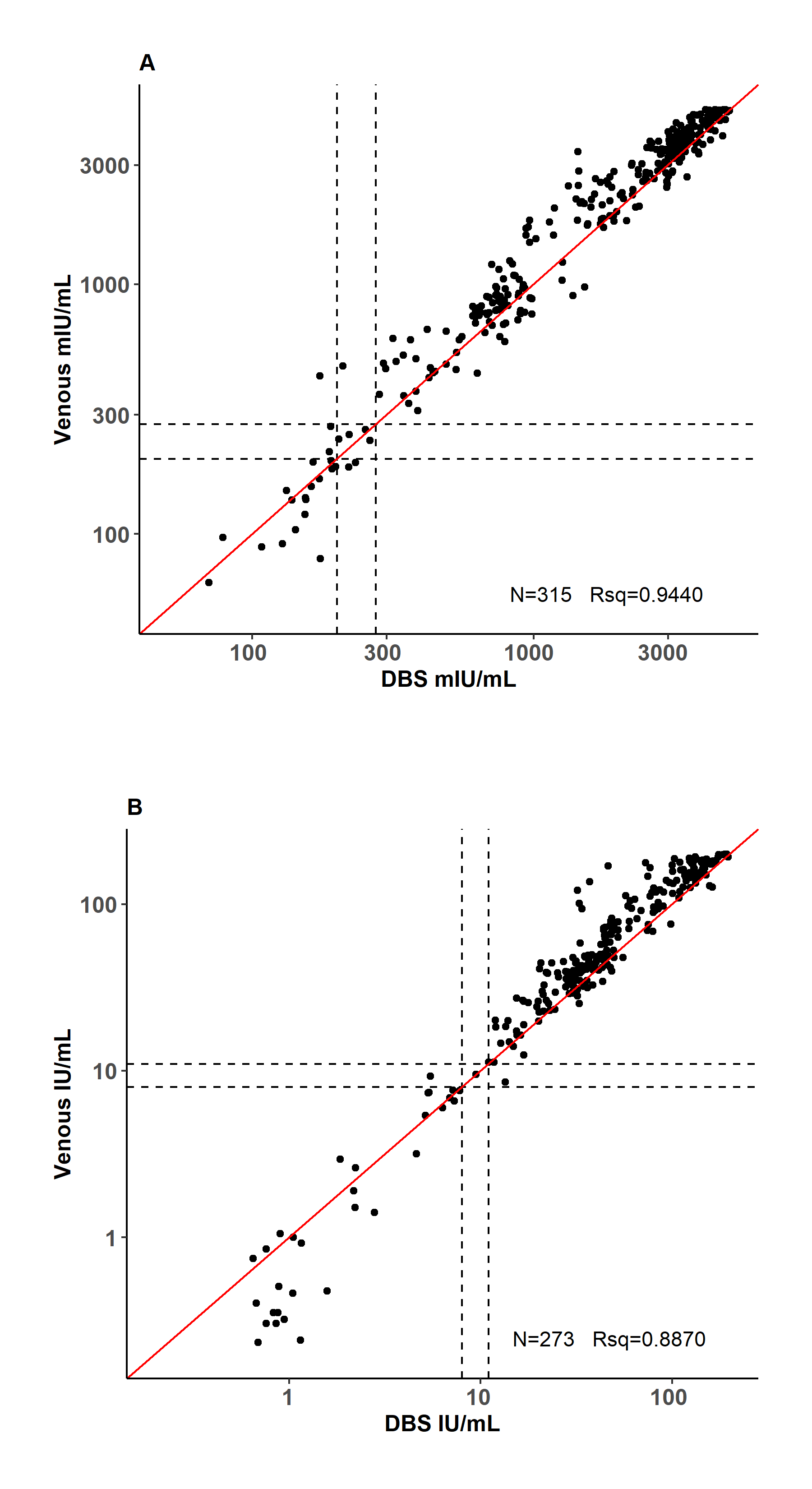

Supplement: FIG S3 [file msphere.01330-20-sf003.tif]
