# Supplementary material for: Diagnostic Accuracy of Dried Blood Spots Collected on HemaSpot HF Devices Compared to Venous Blood Specimens To Estimate Measles and Rubella Seroprevalence
Source: mSphere. 2021 Jul 14;6(4):e01330-20. doi: 10.1128/mSphere.01330-20 (PMC8386429; doi:10.1128/mSphere.01330-20)
Supplement: TABLE S1 [file msphere.01330-20-st001.docx]

| **Dried blood spot specimen** | **Serum specimen** | | | | | | | |  |
| --- | --- | --- | --- | --- | --- | --- | --- | --- | --- |
|  | **Measles** | | | | **Rubella** | | | |  |
|  | Positive | Equivocal | Negative | Total | Positive | Equivocal | Negative | Total |  |
| Positive | 520 | 1 | 0 | 521 | 497 | 1 | 0 | 498 |  |
| Equivocal | 5 | 6 | 3 | 14 | 6 | 1 | 1 | 8 |  |
| Negative | 5 | 4 | 29 | 38 | 7 | 6 | 54 | 67 |  |
| Total | 530 | 11 | 32 | 573 | 510 | 8 | 55 | 573 |  |
| **Diagnostic Accuracy% (95% CI)** | | | | | | | | | |
| % Sensitivity | 98.3 (96.9, 99.2) | | | | 97.5 (95.8, 98.7) | | | |  |
| % Specificity | 90.6 (75.0, 98.0) | | | | 98.2 (90.3, 100) | | | |  |
| % Positive predictive value | 99.4 (98.4, 99.9) | | | | 99.8 (98.9, 100) | | | |  |
| % Negative predictive value | 76.3 (59.8, 88.6) | | | | 80.6 (69.1, 89.2) | | | |  |
